# Physical–psychological–cognitive multimorbidity: a narrative review

**DOI:** 10.3389/fpubh.2026.1851469

**Published:** 2026-07-27

**Authors:** Chunmei Yang, Wei Wei, Wenhui Chai, Bumaryam Abla, Tao Li, Xuan Wang, Hongtao Cai, Juan Tang, Junfang Yang, Ying Liu, Jiao Liu

**Affiliations:** 1 Department of Geriatrics, People's Hospital of Changji Hui Autonomous Prefecture, Changji, Xinjiang, China; 2 Department of Infectious Diseases, People's Hospital of Changji Hui Autonomous Prefecture, Changji, Xinjiang, China; 3 Department of Neurology, People's Hospital of Changji Hui Autonomous Prefecture, Changji, Xinjiang, China

**Keywords:** comprehensive geriatric assessment, epidemiology, healthy aging, pathophysiological mechanisms, physical–psychological–cognitive multimorbidity, risk factors

## Abstract

Physical–psychological–cognitive multimorbidity (PPC-MM) is a distinct phenotype in older adults, defined as the coexistence of chronic physical conditions, psychological disorders, and cognitive impairment. When all three domains are present, the adverse impact on patients may be greater than that associated with single diseases or physical multimorbidity alone. This review delineates operational definitions of PPC-MM across major cohort studies, summarizes global epidemiological characteristics, and synthesizes evidence on multi-level risk factors. Chinese older adults have a PPC-MM prevalence ranging from 5.8% to 17.2%, while European and American populations have a PPC-MM prevalence ranging from 1.2% to 4.3%. These differences may reflect variations in region, population, and diagnostic criteria. Specifically, the definition of physical conditions has been primarily centered on 7–10 disease categories, and psychological disorders are mainly defined by depressive symptoms, and cognitive impairment is assessed using cohort-specific short-form batteries. Low socioeconomic status, adverse childhood experiences, and circadian syndrome have been linked to PPC-MM. Possible mechanisms include hypothalamic–pituitary–adrenal (HPA) axis dysregulation, inflammaging-related immune crosstalk, and loneliness-related feedback loops. PPC-MM reflects interactions across social, psychological, behavioral, and biological factors. Management could move toward integrated care based on comprehensive geriatric assessment, which may help delay functional decline and improve quality of life.

## 1 Introduction

The global population is aging at an accelerated pace, and the burden of chronic diseases among older adults is increasing. A 2025 meta-analysis of 49 studies involving 729,043 older adults reported a global pooled prevalence of multimorbidity of 46.0% (95% CI: 38.0%–55.0%) among individuals aged 60 years and older (1). Traditional multimorbidity research has focused primarily on physical diseases (hypertension, type 2 diabetes, etc.); however, many older adults have not only multiple physical conditions but also psychological disorders or cognitive impairment. Therefore, focusing solely on single physical diseases or physical multimorbidity does not fully capture the complexity of health in older adults.

To address this gap, the concept of physical–psychological–cognitive multimorbidity has gained increasing attention. A 2023 multinational cross-sectional study provided an early systematic investigation of physical–psychological–cognitive multimorbidity and found that individuals with low socioeconomic status had a 12.36-fold higher risk of PPC-MM compared to those with high socioeconomic status (2). The important innovation of PPC-MM is that it explicitly requires the simultaneous presence of three domains: chronic physical condition, psychological disorder and cognitive impairment. PPC-MM differs from traditional multimorbidity in that traditional multimorbidity typically refers to two or more physical diseases (hypertension, type 2 diabetes, coronary heart disease, etc.), whereas PPC-MM adds psychological and cognitive dimensions. This definition is critical because longitudinal evidence indicates that the co-occurrence of physical disease, cognitive impairment, and psychological disorder is associated with a significant synergistic deterioration effect, which may contribute to faster functional decline, increased healthcare utilization, and higher mortality, while dyadic combinations show a much weaker effect (2).

This review aims to summarize and clarify the evolution of operational definitions of PPC-MM across different studies and to summarize its global epidemiological characteristics. It also aims to synthesize multidimensional evidence on sociodemographic factors, early-life experiences, lifestyle, and biological aging to identify key risk factors. Furthermore, it seeks to elucidate the potential pathophysiological network linking the concurrent decline in physical, psychological, and cognitive functions. Finally, it proposes an integrated care strategy grounded in the bio-psycho-social framework to address current challenges in clinical management. These efforts are intended to provide evidence-based support for the early identification of high-risk populations, elucidation of disease mechanisms, and development of precise interventions for PPC-MM.

Literature search and study selection: We searched PubMed, Cochrane Library, Embase, EBSCO MEDLINE Complete, JAMA, CNKI, and Wanfang from database inception to April 2026. Keywords included “physical–psychological–cognitive multimorbidity,” “multimorbidity,” “older adults,” “comprehensive geriatric assessment,” “depressive symptoms,” and “cognitive impairment.” We included cross-sectional studies, prospective and retrospective cohort studies, and systematic reviews published in English or Chinese. Conference abstracts, case reports, editorials, and studies without original data were excluded.

## 2 Definition of PPC-MM

PPC-MM can be regarded as a distinct state in the aging process, characterized by the simultaneous presence of three domains: chronic physical conditions, psychological disorders, and cognitive impairment. Its synergistic adverse effect on health outcomes substantially exceeds that of a single disease or physical multimorbidity alone (3). In specific studies, definitions of PPC-MM impairment still exhibit substantial heterogeneity due to differences in data sources, cultural backgrounds, and assessment tools. These three dimensions are elaborated on separately below:

### 2.1 Definition of physical diseases

The definition of physical diseases has shifted from a focus on core conditions to more comprehensive coverage. Early studies mainly used a standard list of seven core diseases: hypertension, diabetes, cancer, lung disease, heart disease, stroke, and arthritis (4). Barnett used this seven-disease list to study the co-occurrence of physical diseases and mental disorders (5). Clinically, these seven conditions cover several important systems: cardiovascular, metabolic, respiratory, oncologic, and musculoskeletal. Therefore, they provide a reasonably broad reflection of physical multimorbidity. Moreover, these seven diseases are the harmonized core variables shared by major international aging cohort databases (HRS, ELSA, SHARE, KLoSA, CHARLS, MHAS, and LASI). As a result, from 2023 to 2026, the definition of chronic physical conditions in PPC-MM research has been primarily based on these seven diseases.

In cohorts such as those from China, liver and kidney diseases are more common. Therefore, using only the seven core diseases may underestimate the true burden of multimorbidity. To address this, some studies expanded the disease list to nine categories by adding liver and kidney diseases (6). This is especially relevant in older adults, as chronic kidney disease (CKD) patients commonly present with liver disease, osteosarcopenia, and other comorbidities beyond cardiometabolic disorders (7, 8). This expansion improves the completeness of the disease spectrum. However, it may reduce comparability with earlier studies that used only seven diseases. A few studies have attempted a further expansion to 10 categories, adding digestive disorders or osteoporosis to better reflect local disease patterns (9). Nevertheless, a 10-category list has not yet achieved broad consensus. It may also create difficulties for cross-study comparisons because of regional differences. Thus, the nine-category definition is currently the most widely accepted. It balances comprehensive coverage with cross-national comparability.

### 2.2 Definition of psychological disorders

In most PPC-MM studies, psychological disorders are operationalized as clinically significant depressive symptoms and assessed using validated self-rating scales (2, 4, 7). This operationalization reflects the stronger and more consistent association of depression with both physical diseases and cognitive impairment compared to anxiety, as demonstrated in multiple multinational prospective cohorts (2, 4, 6, 7). Different studies select various scales based on data availability and research traditions, but all apply predefined cutoff values as the criterion for classification. The China Health and Retirement Longitudinal Study (CHARLS) uses the 10-item Center for Epidemiologic Studies Depression Scale (CES-D-10) to assess depressive symptoms, with a total score of ≥10 indicating clinically significant depressive symptoms (10). The US Health and Retirement Study (HRS) and the English Longitudinal Study on Aging (ELSA) use the 8-item Center for Epidemiologic Studies Depression Scale (CES-D-8), with a total score of ≥4 as the cutoff value (4). The Survey of Health, Aging and Retirement in Europe (SHARE) uses the EURO-D scale to measure depression, with a cutoff value of ≥4 (2, 4). Research has shown that depressive symptoms reaching a certain level of severity are associated with reduced physical functioning, decreased social participation, and lower treatment adherence. These functional impairments may contribute to the progression of multimorbidity (7, 11).

In recent years, some studies focusing on specific populations have attempted to incorporate anxiety symptoms into the assessment of psychological disorders, aiming to construct a broader category of emotional disorders. These studies use both depression and anxiety scales simultaneously, applying the rule that meeting either criterion is sufficient for classification. In a study on older adults with hypertension in remote areas of China, and this study defined psychological disorders as a Patient Health Questionnaire-9 (PHQ-9) score >4 or a Generalized Anxiety Disorder 7-item scale (GAD-7) score >4 (12). Some studies also adopted the same dual-scale strategy in studies on older adults with hypertension and diabetes in rural areas (9, 13). Notably, all three studies focused on rural populations or specific disease groups in China, and their study designs reflect a more comprehensive assessment of the mental health status of these populations. These studies broaden the definition of psychological disorders.

However, multiple multinational cohort studies indicate that depressive symptoms are more strongly associated with physical diseases and cognitive impairment than anxiety (11, 14). A multinational prospective cohort study has shown that the coexistence of depressive symptoms with physical disorders significantly increases the risk of dementia and accelerates cognitive decline independently of cardiometabolic multimorbidity (6, 7). In the pathophysiological mechanisms of multimorbidity, depression is hypothesized to be a key link between physical and cognitive decline (14). Anxiety often occurs as a comorbid condition with depression, and its independent predictive value for multimorbidity trajectories remains unclear once depressive symptoms are taken into account (7, 14). Therefore, the emphasis on depressive symptoms in the operationalization of psychological disorders is empirically justified in the PPC-MM literature.

### 2.3 Definition of cognitive impairment

Cognitive function refers to the mental processes involved in acquiring, processing, storing, and retrieving information. It encompasses multiple domains, including episodic memory, attention, executive function, language, and visuospatial ability (15, 16). Cognitive impairment refers to varying degrees of functional impairment in one or more cognitive domains resulting from various causes. Based on its severity, cognitive impairment is categorized into mild cognitive impairment (MCI) and dementia (16). Large cohort studies cannot implement comprehensive neuropsychological assessments. Therefore, most studies adopt short-form neuropsychological task batteries combined with cohort-specific cutoff values. Considerable heterogeneity has been associated with definitions across studies.

The cognitive assessment in the China CHARLS cohort primarily comprises two components: episodic memory and mental integrity. Episodic memory is evaluated through immediate and delayed word recall tests, while mental integrity encompasses tasks such as temporal orientation, figure drawing, and serial subtraction (6, 17). The US HRS and UK ELSA cohorts employ similar assessment protocols, including immediate and delayed word recall, serial subtraction, and countdown tasks. With a total score ranging from 0 to 27, respondents are classified as having dementia (scores 0–6), mild cognitive impairment (scores 7–11), or normal cognition (scores 12–27) (18). The cognitive assessment in the European SHARE cohort includes tests of episodic memory and verbal fluency. Studies generally use a cutoff of 1.5 standard deviations below the age-specific mean to define cognitive impairment; impairment in either domain is classified as the presence of cognitive impairment (2, 4, 7).

Some studies adjust cutoff values by educational level. For example, on the Mini-Mental State Examination (MMSE), the cutoff values are ≤17 for illiterate individuals, ≤20 for those with primary school education, and ≤24 for those with junior high school education or above (13). However, because of its relatively long administration time, the MMSE is often used only for sensitivity analyses or subsample validation in large-scale epidemiological surveys. In addition, the 8-item Ascertain Dementia questionnaire (AD8) uses a score of ≥2 as the cutoff for cognitive impairment (7), and the Spanish version of the Montreal Cognitive Assessment (MoCA) has been validated with a cutoff score of 21 (19); both have been used as supplementary tools in some studies.

Overall, current cognitive assessment approaches form a multidimensional, complementary system that includes cohort-specific batteries and standardized tools (MMSE, MoCA, AD8). Despite varying cutoffs, these methods enable early identification and staging of cognitive impairment. Future studies may combine tools to improve accuracy and applicability across populations.

Traditional multimorbidity is typically defined as the coexistence of two or more chronic physical conditions, it does not include psychological or cognitive disorders. Frailty is defined as an increased vulnerability to stressors in the context of a decline in physiological reserve across multiple systems. Clinically, frailty may manifest as weight loss, exhaustion, reduced grip strength, slow gait speed, and low physical activity (20). Frailty primarily concerns physical function (21). Different from both of these, the core definition of PPC-MM is the simultaneous coexistence of physical, psychological, and cognitive disorders.

Although the coexistence of physical, psychological, and cognitive disorders is the core definition of PPC-MM, substantial heterogeneity remains across studies. This heterogeneity is seen in the selection of physical disease categories, the inclusion of anxiety in psychological disorders, and the criteria for defining cognitive impairment. These inconsistencies in definitions, together with the diversity of assessment methods, point to the need for a standardized PPC-MM assessment framework. Cross-national and cross-population validation studies are also needed in future research.

## 3 Epidemiological burden

The global burden of PPC-MM is substantial. In a multinational cross-sectional study covering 33 countries, the prevalence of multimorbidity was 24.5% in high-income countries, 33.9% in upper-middle-income countries, and 8.1% in lower-middle-income countries (2). Another study by Lin L and colleagues reported prevalence rates of 29.1% for physical–cognitive multimorbidity, 21.7% for physical–psychological multimorbidity, 18.5% for psychological–cognitive multimorbidity, and 17.2% for physical–psychological–cognitive multimorbidity (7).

The study by Feng reported that the prevalences of physical, psychological, cognitive, physical–psychological multimorbidity, physical–cognitive multimorbidity, psychological–cognitive multimorbidity, and physical–psychological–cognitive multimorbidity were 63.55, 30.12, 64.55, 22.31, 42.03, 22.57, and 16.74%, respectively (12). The study by Bai reported that the prevalence of physical comorbidity was 74.9%, psychological comorbidity 32.5%, cognitive comorbidity 29.7%, physical–psychological comorbidity 26.1%, physical–cognitive comorbidity 22.9%, psychological–cognitive comorbidity 11.7%, and physical–psychological–cognitive comorbidity 9.8% (13). In the study described the prevalence of PPC-MM was 5.8% (17). Based on the above studies, Chinese older adults have a PPC-MM prevalence ranging from 5.8% to 17.2%, while European and American populations have a PPC-MM prevalence ranging from 1.2% to 4.3%.

Relevant studies have shown that lower educational level, lower household wealth, and lower composite socioeconomic status scores are significantly associated with multimorbidity in high-income and upper-middle-income countries. In high-income countries, the odds ratio (OR) for physical–psychological–cognitive multimorbidity in individuals with low versus high socioeconomic status was 12.36 (95% CI: 10.29–14.85; *p* < 0.0001); in upper-middle-income countries, the corresponding OR was 23.84 (95% CI: 18.85–30.14; *p* < 0.0001); whereas in the lower-middle-income country (India), the associations were weaker for some outcomes and reversed for others (2).

Moreover, participants with both low educational level and low household wealth had the highest odds of multimorbidity: in high-income countries, the OR for PPC-MM was 21.21 (95% CI: 15.95–28.19; *p* < 0.0001); in upper-middle-income countries, it was 37.07 (95% CI: 25.66–53.56; *p* < 0.0001); and in the lower-middle-income country, it was 54.96 (95% CI: 7.66–394.38; *p* < 0.0001) (2). In addition to educational level and economic status, age was identified as a significant factor associated with the prevalence of multimorbidity, with higher prevalence observed among participants aged 81–90 years and ≥91 years (1). An analysis showed that the prevalence of multimorbidity was higher in females (48%) than in males (41%) (1). A study found that the prevalence of cognitive impairment was significantly higher in females than in males in the rural cohort (15). Table 1 summarizes the prevalence estimates and key cohort characteristics of the included studies.

**Table 1:** Prevalence and cohort characteristics of PPC-MM in included studies.

Author, year	Area	Time of survey	Study design	Sample size (*N*)	Age	Population/source	PPC-MM definition	PPC-MM prevalence (%)	OR (95% CI) for PPC-MM	References
Ni Y, 2023 (2)	33 countries (China, South Korea, US, England, Europe, Mexico, India)	2017–2020	Cross-sectional	167,376	≥45 years	Multi-country cohorts (HRS, ELSA, SHARE, KLoSA, CHARLS, MHAS, LASI)	Physical: ≥1 of 7 self-reported chronic conditions; Psychological: study-specific depression scale; Cognitive: study-specific cognitive test; PPC-MM = co-occurrence of all three	Not reported	Low vs. high SES: HIC 12.36 (10.29–14.85); UMIC 23.84 (18.85–30.14)	(2)
Lin L, 2025 (7)	China	September 2024–December 2024	Cross-sectional	437	≥60 years	Community-dwelling older adults	Physical: ≥1 physician-diagnosed chronic disease; Psychological: PHQ-9 ≥ 5; Cognitive: AD-8 ≥ 2; PPC-MM = meeting all three	17.2%	Multivariate logistic regression identified long-term medication use (OR = 3.24, 95% CI [1.28–8.21]), higher multimorbidity burden (OR = 7.31, 95% CI [3.27–16.36]), social frailty (OR = 3.49, 95% CI [1.74–7.01]), and lower EQ-5D scores (OR = 0.07, 95% CI [0.02–0.26]) as significant predictors of PPC status	(7)
You Y, 2025 (22)	China, Europe, the US, and the UK	Baseline: 2014 (CHARLS), 2015 (HRS), 2017 (SHARE), 2006 (ELSA); Outcome: 2022	prospective cohorts	CHARLS: 10,433; HRS: 4,648; SHARE: 30,107; ELSA: 2,694	CHARLS: ≥45 years; HRS, ELSA, SHARE: ≥50 years	Multi-country aging cohorts (CHARLS, HRS, SHARE, ELSA)	Physical: ≥1 of 7 chronic conditions; Psychological: cohort-specific depression scale; Cognitive: cohort-specific cognitive tests; PPC-MM = co-occurrence of all three	China 8.4%, US 1.2%, Europe 3.0%, UK 4.3%	Not reported	(22)
Zhou Y, 2024 (11)	24 countries (United States, Europe, Asia)	2002–2021	Prospective cohort	20,250	≥45 years	HRS, ELSA, SHARE, CHARLS, KLoSA	Physical: ≥1 of 7 chronic conditions; Psychological: study-specific depression scale (CES-D, EURO-D); Cognitive: study-specific cognitive tests; PPC-MM = co-occurrence of all three	7.4%	Not reported	(4)
Zhou Y, 2025 (23)	US, England, and China	2011–2020	Prospective cohort	24,955	≥45 years	HRS, ELSA, CHARLS	Physical: ≥1 of 7 chronic conditions; Psychological: CES-D (HRS/ELSA ≥4, CHARLS ≥10); Cognitive: study-specific tests (>1.5 SD below age mean); PPC-MM = three patterns	11.3%	Not reported	(23)
Zhang T, 2025 (17)	China	2014–2015	Nationwide population-representative survey	11,124	≥45 years	CHARLS	Physical: ≥1 of 7 self-reported chronic diseases; Depressive: CES-D-10 score ≥10; Cognitive: Telephone Interview for Cognitive Status (TICS) score indicating cognitive impairment; PPC-MM = co-occurrence of all three	5.8%	The estimated odds ratios (OR) was 2.91 (2.15–3.96) for PPC-MM	(17)
Feng Y, 2025 (12)	China	July 1–August 31, 2023	Cross-sectional	17,728	≥65 years	Community-dwelling older adults with hypertension from remote rural China	Physical: ≥1 of 19 chronic conditions; Psychological: PHQ-9 > 4 or GAD-7 > 4; Cognitive: MMSE score <25.6; PPC-MM = co-occurrence of physical + psychological + cognitive disorders	16.74%	Five healthy LBs vs. none: 0.13 (0.07–0.23)	(12)
Miao Y, 2024 (9)	China	July 1–August 31, 2023	Cross-sectional	18,447	≥65 years	Older adults with hypertension in rural China	Physical: ≥1 of 9 self-reported chronic conditions; Psychological: PHQ-9 > 4 or GAD-7 > 4; Cognitive: MMSE score below education-specific cutoff; PPC-MM = co-occurrence of all three	Not reported (the multimorbidity prevalence: women 38.67%, men 30.64%)	Not reported	(9)
Bai M, 2025 (13)	China	July 1–August 31, 2023	Cross-sectional	6,057	≥65 years	Older adults with diabetes in rural China (from National Basic Public Health Database)	Physical: ≥1 of 4 chronic disease categories; Psychological: PHQ-9 or GAD-7 score >5; Cognitive: MMSE score below education-specific cutoffs; PPC-MM = co-occurrence of all three	9.8%	A one-unit increase in the lifestyle score was associated with a 34% (OR: 0.66, 95% CI: 0.61–0.72) reduction in the odds of PPC-MM	(13)
Talifu Z, 2025 (25)	United Kingdom (UK Biobank)	Baseline: 2006–2010; Follow-up: until 2022 (median 13.6 years)	Prospective cohort	332,012	37–73 years	UK Biobank participants	Physical: ≥1 of 35 long-term conditions; Psychological: substance use disorder, schizophrenia, mood disorders, etc.; Cognitive: Alzheimer’s disease, other dementias, or cognitive test impairment. PPC-MM = co-occurrence of all three	3.2%	Not reported	(25)
Bi J, 2025 (24)	China	Baseline: 2011; Follow-up: 2013, 2015, 2018	Prospective cohort	8,262	≥45 years	CHARLS	Physical: ≥1 of 7 self-reported chronic diseasesa; Psychological: CES-D-10 score ≥10; Cognitive: score ≥1.5 SD below age-specific mean in any domain of immediate/delayed word recall, serial-7 subtraction, or temporal orientation; PPC-MM = co-occurrence of all three	Not reported	HR (95% CI) for CircS vs. no CircS: 1.48 (1.09–2.02)	(24)
Xinwei Z, 2025 (10)	China	2011–2018	Longitudinal cohort study	6,843	≥45 years	CHARLS	Physical: ≥1 of 7 chronic diseases, psychological (CESD-10 ≥ 10), cognitive (score>1.5 SD below age-specific mean in any of immediate recall, delayed recall, serial 7 s, orientation), PPC-MM = co-occurrence of three diseases	Not reported	Low SWB is associated with approximately a 2-fold increased risk of PPC-MM. Main analysis (discrete-time model): *H* = 2.03 (95% CI: 1.82–2.26); Time-varying MSM: *H* = 1.95 (1.73–2.19); Competing-risk (Fine-Gray) model: sHR = 1.26 (1.12–1.40)	(10)

HRS, US Health and Retirement Study; ELSA, English Longitudinal Study on Aging; KLOSA, Korean Longitudinal Study of Aging; SHARE, Survey of Health, Aging and Retirement in Europe; CHARLS, China Health and Retirement Longitudinal Study; MHAS, Mexican Health and Aging Study; LASI, Longitudinal Aging Study in India; CES-D/CESD-10, Center for Epidemiologic Studies Depression Scale; PHQ-9, Patient Health Questionnaire-9; GAD-7, Generalized Anxiety Disorder 7-item scale; MMSE, Mini-Mental State Examination; AD-8, Ascertain Dementia 8-item questionnaire; TICS, Telephone Interview for Cognitive Status; EQ-5D, EuroQol 5-dimension questionnaire; CircS, Circadian syndrome; SWB, Subjective wellbeing; MSM, Marginal structural model; SES, Socioeconomic status; HIC, High-income countries; UMIC, Upper-middle-income countries; LB, Lifestyle behavior.

PPC-MM has become a serious global public health challenge. Its prevalence is significantly influenced by socioeconomic status, age, and sex, with the highest risk observed in low-resource populations, older adults, and females. Future prevention and control strategies should go beyond a purely biomedical perspective and focus on social determinants of health, implementing early screening and comprehensive interventions for vulnerable populations to effectively reduce this growing disease burden.

## 4 Risk factors and pathophysiological mechanisms

The development and progression of PPC-MM are not attributable to a single factor, but rather involve the accumulation and interaction of social, psychological, behavioral, and biological factors across the life course. This section reviews the key factors and underlying mechanisms from four dimensions: social determinants, lifestyle factors, biological aging, and psychological mediators.

### 4.1 Social determinants and early-life experiences

Socioeconomic status is the most stable structural factor associated with PPC-MM. Cross-sectional studies have shown that, across high-income, upper-middle-income, and lower-middle-income countries, individuals with lower socioeconomic status had more than 10 times the odds of PPC-MM compared to those with higher socioeconomic status. This gradient was steeper in lower-income countries (2). This marked social inequality is not merely a reflection of disparities in healthcare access. Its deeper cause is the accumulation of chronic stress in the context of prolonged exposure to adverse environments (22, 23).

Studies from a life-course perspective further reveal that adverse childhood experiences have profound impacts on health in later life (17, 22, 23). After adjusting for age, sex, and educational level, each additional stressful life event (SLE) in childhood was associated with a 1.29-fold increase in the risk of PPC-MM (HR = 1.29, 95% CI: 1.22–1.35), and each additional SLE in adulthood was associated with a 1.35-fold increase (HR = 1.35, 95% CI: 1.29–1.41). Individuals who experienced SLEs in both childhood and adulthood had a 1.89-fold higher risk of PPC-MM (HR = 1.89, 95% CI: 1.69–2.11) (23).

Studies have further distinguished two types of early-life adversity. Deprivation is primarily associated with overall multimorbidity risk, whereas threat is more specifically associated with patterns involving psychological disorders (22). The cumulative effects of major loss in childhood and stressful life events in adulthood may contribute to dysregulation of cortisol circadian rhythms and glucocorticoid resistance in the context of sustained activation of the HPA axis (14, 23). Such neuroendocrine dysregulation may promote abdominal fat accumulation and insulin resistance, and may also directly damage hippocampal neurons. This process may create a positive stress-inflammation feedback loop, forming an early biological basis for multisystem instability (23).

### 4.2 Lifestyle and circadian disruption

Unhealthy lifestyles and circadian disruption are considered key links between external environmental stress and internal biological dysregulation. A study focusing on older adults with diabetes in rural China found that smoking, alcohol consumption, and physical inactivity significantly increased the risk of PPC-MM, whereas adherence to multiple healthy lifestyle behaviors reduced this risk (13). In recent years, circadian syndrome (CircS) has emerged as a potential upstream factor. This syndrome is characterized by the disruption of synchronization among sleep–wake cycles, metabolic rhythms, and mood rhythms. A prospective cohort study based on CHARLS showed that, during a median follow-up of 7 years, baseline CircS was significantly associated with an increased risk of incident PPC-MM (HR = 1.48, 95% CI: 1.09–2.02) and psychological–cognitive multimorbidity (HR = 1.38, 95% CI: 1.06–1.79) after full adjustment (24). A significant dose–response relationship was also observed. The population attributable fraction of CircS for PPC-MM was 16.8%. These associations were more pronounced in women and in participants without chronic diseases at baseline (24).

Possible mechanisms include dysregulation of core clock genes, which may impair insulin signaling and mitochondrial function. Fragmented nighttime sleep may interfere with memory consolidation and reduce daytime activity. These changes may translate external psychosocial stress into internal multisystem decoupling and decline, which may be associated with synergistic deterioration of physical, psychological, and cognitive functions under circadian disruption.

### 4.3 Biological aging and inflammatory network

As the disease progresses, accelerated biological aging and chronic low-grade inflammation become key pathological processes associated with the worsening of PPC-MM (25, 26). A study based on the UK Biobank showed that, for triple comorbidity, each standard deviation increase in Klemera–Doubal Method biological age (KDM-BA) acceleration was associated with a 20% increase in the odds of the condition (OR = 1.20, 95% CI: 1.15–1.26, *p* < 0.001), while each standard deviation increase in PhenoAge acceleration was associated with a 12% increase (OR = 1.12, 95% CI: 1.07–1.17, *p* < 0.001) (25). The study examined a range of lifestyle, social, psychological, cognitive, and physical factors as potential mediators, with all analyses stratified by sex. Causal mediation analyses were used to estimate indirect effects in single-mediator analyses. Statistically significant mediators were then included in multiple-mediator analyses to examine their combined effects. The results showed that both older men and women with frailty had higher odds of mortality after 6 years compared to those without frailty (26). Future studies may further explore the mediating role of frailty syndrome, as a clinical phenotype of biological aging, in the association between PPC-MM and mortality risk.

Research suggests that cardiometabolic diseases and depression may jointly contribute to accelerated cognitive decline through shared mechanisms, including inflammatory pathways, neuroendocrine dysregulation, and vascular dysfunction (14, 25, 27). This accelerated aging is often accompanied by a state of inflammaging characterized by persistently elevated levels of pro-inflammatory cytokines such as C-reactive protein (CRP) and interleukin-6 (IL-6) (14, 25), and increased production of reactive oxygen species (ROS), which collectively contribute to oxidative stress and inflammaging. These inflammatory mediators can cross the blood–brain barrier via peripheral-central immune pathways, activate microglia, interfere with synaptic plasticity, and inhibit neurotrophic factors, thereby contributing to anhedonia, fatigue, and cognitive decline (14, 25). Future research aimed at elucidating the molecular basis of neuroinflammatory mechanisms in PPC-MM may provide direct evidence for how peripheral inflammatory signals cross the blood–brain barrier and reshape central neural circuits.

### 4.4 The psychosocial mediating role of loneliness

Loneliness plays a key psychosocial mediating role in the complex network of PPC-MM (14). A national study of Chinese adults found that loneliness significantly mediated the association between adverse childhood experiences and physical-depressive-cognitive multimorbidity, with mediation proportions ranging from 8.7% to 32.5%. This mediation analysis suggested that these associations were partially mediated by loneliness, with evidence indicating that loneliness can trigger stress responses, elevate blood pressure, and impair the immune system through inflammatory and hormonal changes (17). Furthermore, a multicohort study revealed that social inactivity is associated with a worsening of the combined impact of cardiometabolic multimorbidity on physical, psychological, and cognitive function (28, 29).

Several possible explanations have been proposed. Notably, loneliness has been associated with changes in cellular function and increased vascular resistance, which may elevate the risk of stroke, hypertension, depression, and cognitive decline (14). Some scholars have suggested that the shared biological pathways between depression, the biopsychosocial frailty phenotype, and late-life cognitive disorders may involve inflammatory and cardiometabolic processes, as well as multimorbidity, loneliness, mitochondrial dysfunction, dopaminergic neurotransmission, specific personality traits, lack of subjective or objective social support, and neuroendocrine dysregulation (14). The above findings indicate complex interactions between loneliness, sleep disturbances, and elevated inflammation (14, 17). This dual psycho-biological impact may contribute to a negative feedback loop of loneliness, functional decline, and social avoidance, which may be associated with the mutually reinforcing deterioration of physical, psychological, and cognitive health (14, 30).

**Figure 1:**
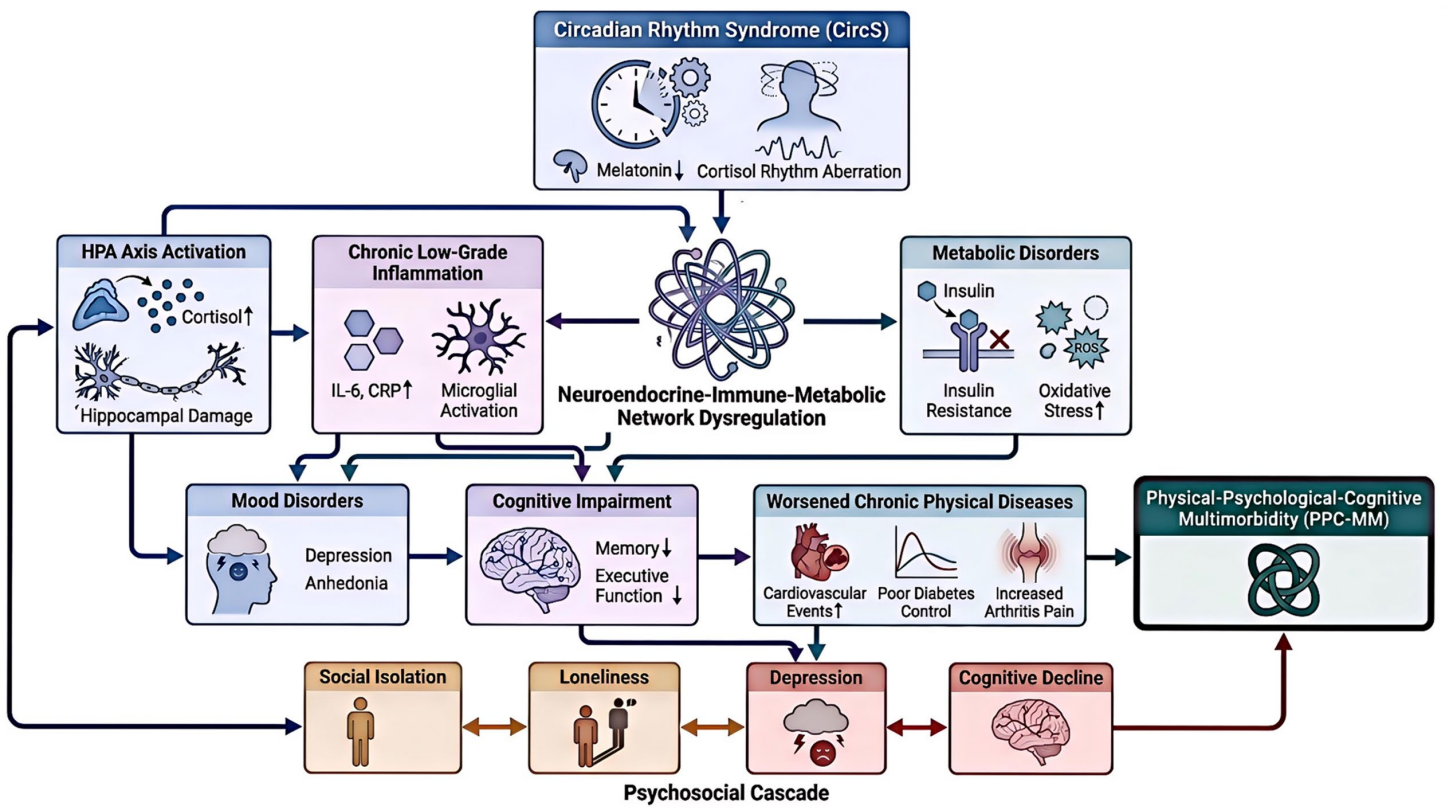
Pathophysiological network underlying physical–psychological–cognitive multimorbidity (PPC-MM). For simplicity, bidirectional arrows are shown only for loneliness, depression, and cognitive decline. Inflammation also has bidirectional links with these domains.

Based on the above, we integrated the key risk factors and pathophysiological mechanisms underlying the development and progression of PPC-MM. As illustrated in Figure 1, social determinants and psychosocial factors act on the body through multiple pathways, including activation of the HPA axis, induction of chronic inflammation and biological aging, and disruption of circadian rhythms, and may contribute to the synergistic deterioration of physical, psychological, and cognitive functions. These mechanisms do not operate independently but are interconnected and mutually reinforcing, forming a self-perpetuating pathological network.

## 5 Discussion

### 5.1 Current limitations and challenges

The intertwining of physical, psychological, and cognitive symptoms in PPC-MM presents significant challenges. Current research limitations primarily stem from several factors. First, a reliance on self-reported data often lacks validation against clinical diagnostic gold standards. Second, substantial heterogeneity across studies in disease definitions, assessment tools, and cutoff values restricts data integration and meta-analyses (1, 2). Moreover, the predominance of cross-sectional designs makes it difficult to elucidate the temporal relationships between risk factors and the onset of multimorbidity; in particular, the bidirectional causal mechanisms between depression and multimorbidity remain unclear (10, 11). Although some longitudinal studies suggest long-term impacts of socioeconomic status and childhood adversity, the strength of causal inference remains insufficient (22, 23). Notably, there is also a paucity of randomized controlled trials (RCTs) specifically targeting the PPC-MM phenotype, and existing interventions largely focus on single dimensions or solely on physical multimorbidity, failing to evaluate the composite efficacy of integrated strategies.

### 5.2 Clinical implications and future directions

Given the complexity of PPC-MM, traditional disease-centric models face multiple dilemmas. Fragmentation across specialties increases the risk of polypharmacy and overlooks drug–drug interactions (6). In older adults, psychological and cognitive impairments are frequently misidentified as normal aging, which may be associated with underdiagnosis (14). Additionally, cognitive decline compromises self-management capabilities, severely affecting treatment adherence. Consequently, establishing interdisciplinary collaborative teams and implementing patient-centered, goal-oriented care has become a consensus (14, 31). In multidisciplinary team (MDT) management, Comprehensive Geriatric Assessment (CGA) should serve as the core tool for comprehensive medication review, screening for psychological and cognitive impairments, and assessing social support (31). Furthermore, interventions must be coupled with structural policies to mitigate socioeconomic inequalities (2, 7) and leverage community resources to alleviate loneliness and regulate circadian rhythms (17, 24). Given that a lack of disease awareness directly negatively impacts preventive behaviors, future care protocols should prioritize improving patient awareness of multimorbidity to optimize long-term management outcomes through enhanced self-efficacy.

## 6 Conclusion

Older adults are a high-risk population for PPC-MM. This review clearly elucidates that PPC-MM is not a random clustering of diseases but a systemic functional decline associated with the interaction of social, psychological, behavioral, and biological factors across the life course. These findings suggest that clinical prevention and control must transcend single-disease thinking and shift toward an integrated care model centered on comprehensive geriatric assessment. Future research may focus on the neuroinflammatory mechanisms to further elucidate the molecular basis of PPC-MM. Future directions should also include establishing standardized definitions, developing CGA-guided integrated interventions, and constructing risk prediction models for PPC-MM. These efforts may help provide precise intervention targets to block the cascading deterioration of PPC-MM, which could contribute to delaying functional decline and improving quality of life in older adults.
